# A solitary brain metastasis as the only site of recurrence of HR positive, HER2 negative breast cancer: a case report and review of the literature

**DOI:** 10.1186/s13256-020-02615-2

**Published:** 2021-01-07

**Authors:** Sandipkumar H Patel, Yoshihito David Saito, Zaibo Li, Bhuvaneswari Ramaswamy, Andrew Stiff, Mahmoud Kassem, Robert Wesolowski

**Affiliations:** 1 Division of Medical Oncology, The Ohio State University Wexner Medical Center, 1310D Lincoln Tower, 1800 Cannon Dr., Columbus, OH 43210 USA; 2 The Ohio State University Comprehensive Cancer Center, The Ohio State University, 410 W 12th Avenue, Columbus, OH 43210 USA; 3 Department of Pathology, The Ohio State University, 410 W 10th AveN337B Doan Hall, Columbus, OH 43210-1267 USA; 4 The Queen’s Medical Center - West Oahu Queen’s Cancer Center - West Oahu, Sullivan Care Center, 91-2127 Fort Weaver Road, Ewa Beach, HI 96706 USA

**Keywords:** Breast cancer, Brain metastases, Chemotherapy, HER2, Hormone receptors

## Abstract

**Background:**

Breast cancer is one of the most common causes of brain metastases. However, the presence of isolated central nervous system (CNS) metastatic disease early in the course of disease relapse is a rare event in cases of hormone receptor positive, human epidermal growth factor receptor 2 (HER2) negative breast cancer.

**Case presentation:**

We summarize the clinical course of a pre-menopausal, 39-year old Caucasian female with history of operable, hormone receptor positive, HER2 negative breast cancer who was initially treated with curative-intend therapy but who unfortunately developed solitary metastatic lesion in the left thalamus. A biopsy of the lesion confirmed the presence of hormone receptor positive, HER2 negative metastatic breast cancer. Patient’s CNS metastases continued to progress without any evidence of metastatic disease outside of the central nervous system and she eventually passed away about 5 years after the date of her initial diagnosis and 18 months following the diagnosis with brain metastasis.

**Conclusion:**

Based on our case, although rare, patients with treated, operable, hormone receptor positive, HER2 negative breast cancer can present with solitary brain metastasis as the only sign of disease recurrence.

## Introduction

Brain metastases from breast cancer occur in 15–25% of patients, representing the second most common cancer to metastasize to the brain, after lung cancer [1]. Unfortunately, patients who develop brain metastases tend to have poor prognosis with short overall survival. The most common subsets of patients who experience brain metastases are those with triple negative breast cancer (TNBC) and human epidermal growth factor receptor 2 (HER2) positive disease. In particular, approximately 50% of patients with stage IV, HER2 positive breast cancer experience metastatic disease in the brain at some point of their disease trajectory [2]. The rising incidence of brain metastases in HER2 positive and TNBC may be due in part to the development of novel more effective systemic therapies leading to longer survival [3]. For example, meta-analysis of randomized trials testing addition of anti-HER2 antibody trastuzumab to standard adjuvant chemotherapy revealed higher rates of central nervous system (CNS) metastasis in patients treated with trastuzumab, despite reduction of the total number of extra-cranial relapses [4]. In general, brain metastasis from hormone receptor (HR) positive, HER2-negative breast cancer are less common and appear later in the course of the disease compared to HER2 positive and TNBC subtypes [5, 6].

Although the incidence of brain metastases in breast cancer is increasing, the finding of CNS involvement in the absence of other extra-cranial disease in patients with HR positive, HER2 negative breast cancer, is exceedingly rare. Here, we report a case of a pre-menopausal female with HR positive, HER2 negative breast cancer who developed brain metastases as the only site of disease recurrence. Despite progression in the CNS resulting in eventual death, no radiographic signs of extra-cranial metastatic disease was evident. We believe that this case illustrates a very rare case of a patient with HR positive, HER2 negative breast cancer who developed solitary metastatic deposit as the site of the first recurrence of breast cancer. In addition, the location of the oligometastatic disease in thalamus is not common (most brain metastases from breast cancer are supratentorial). The purpose of our case report is to pay attention to careful follow-up of patients with HR positive, HER2 negative breast cancer for unapparent metastasis.

## Case presentation

**Fig. 1:**
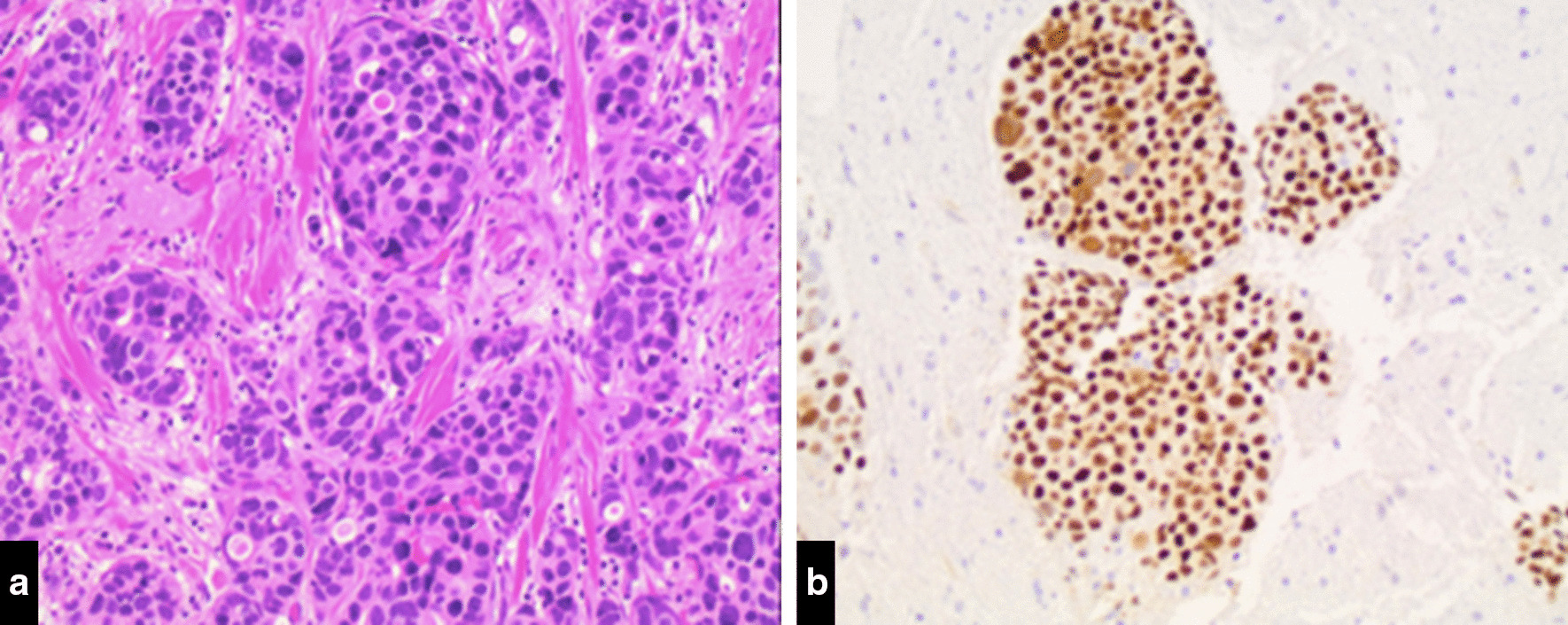
**a** Primary invasive ductal carcinoma (left breast lesion, core needle biopsy). Hematoxylin & Eosin (H & E) stain, ×50. **b** Expression of estrogen receptors (left breast lesion, core needle biopsy), immunohistochemical (IHC) stain, ×50.

**Fig. 2:**
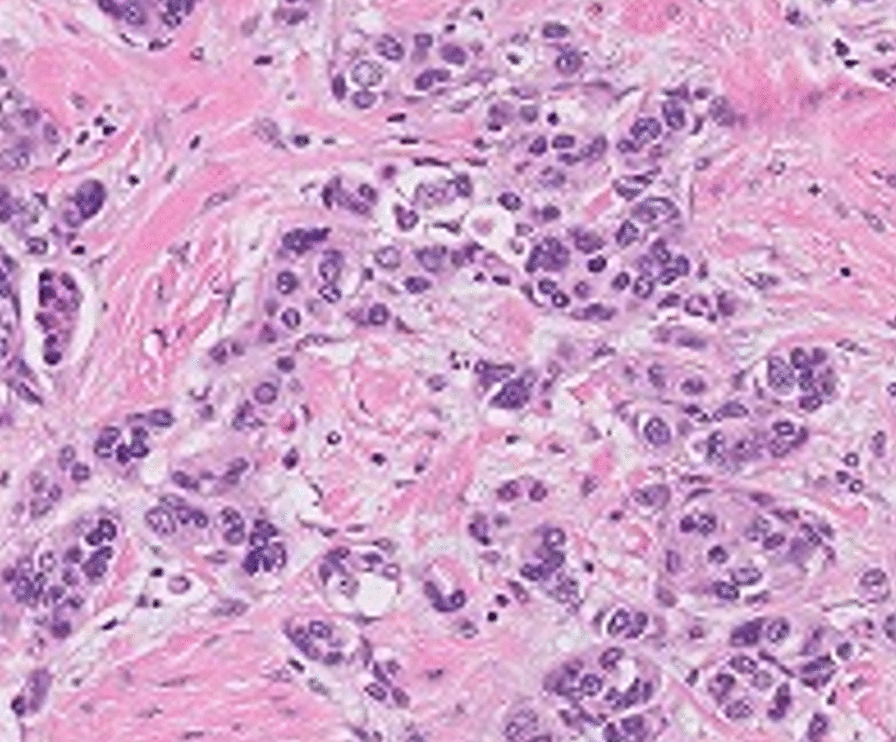
Primary invasive ductal carcinoma (left breast, mastectomy). Hematoxylin & Eosin (H & E) stain, ×50.

The patient was a 39-year-old pre-menopausal Caucasian female with otherwise an unremarkable medical history who initially palpated a lump in her left breast approximately 4-months before presenting to the clinic for evaluation in May, 2011. She had no cancer history in her family. Her diagnostic assessment included (1) a left breast ultrasound which showed a macro-lobulated mass with Doppler positive blood flow and internal echoes measuring 3.4 × 2.3 × 2.6 cm, (2) core needle biopsy of the lesion showed a grade 3 invasive ductal carcinoma (Fig. 1a) that was estrogen receptor (ER) (Fig. 1b) and progesterone receptor (PR) positive (100% and 10% positivity, respectively) and HER2 negative by  fluorescence in situ hybridization (HER2/CEN17 ratio = 1.0). There were no lymphovascular invasion or ductal carcinoma in situ component. Additionally, ultrasound-guided biopsy of an enlarged palpable axillary lymph node was also positive for metastatic carcinoma. In mid-June 2011, the patient initiated neo-adjuvant chemotherapy with 4 cycles of dose-dense doxorubicin and cyclophosphamide followed by 1 cycle of paclitaxel because she has clinical stage IIb (T2N1M0) breast cancer. She subsequently received 3 cycles of docetaxel due to a paclitaxel shortage. Following completion of neo-adjuvant chemotherapy, she underwent left mastectomy and axillary lymph node dissection in late 2011. Surgical pathology of the left breast revealed invasive ductal carcinoma measuring 1.2 cm in the largest diameter which had therapy related cytopathic effect (Fig. 2). Complete axillary node dissection was done and revealed that seven of eleven axillary lymph nodes were involved with metastatic breast carcinoma and the patient had pTNM staging of ypT1c pN2. All surgical margins were negative. She then completed standard adjuvant radiation therapy and was started on Tamoxifen. Additionally, the patient  underwent genetic testing that revealed no detrimental germline mutations in *BRCA1* and *BRCA2* genes.

**Fig. 3:**
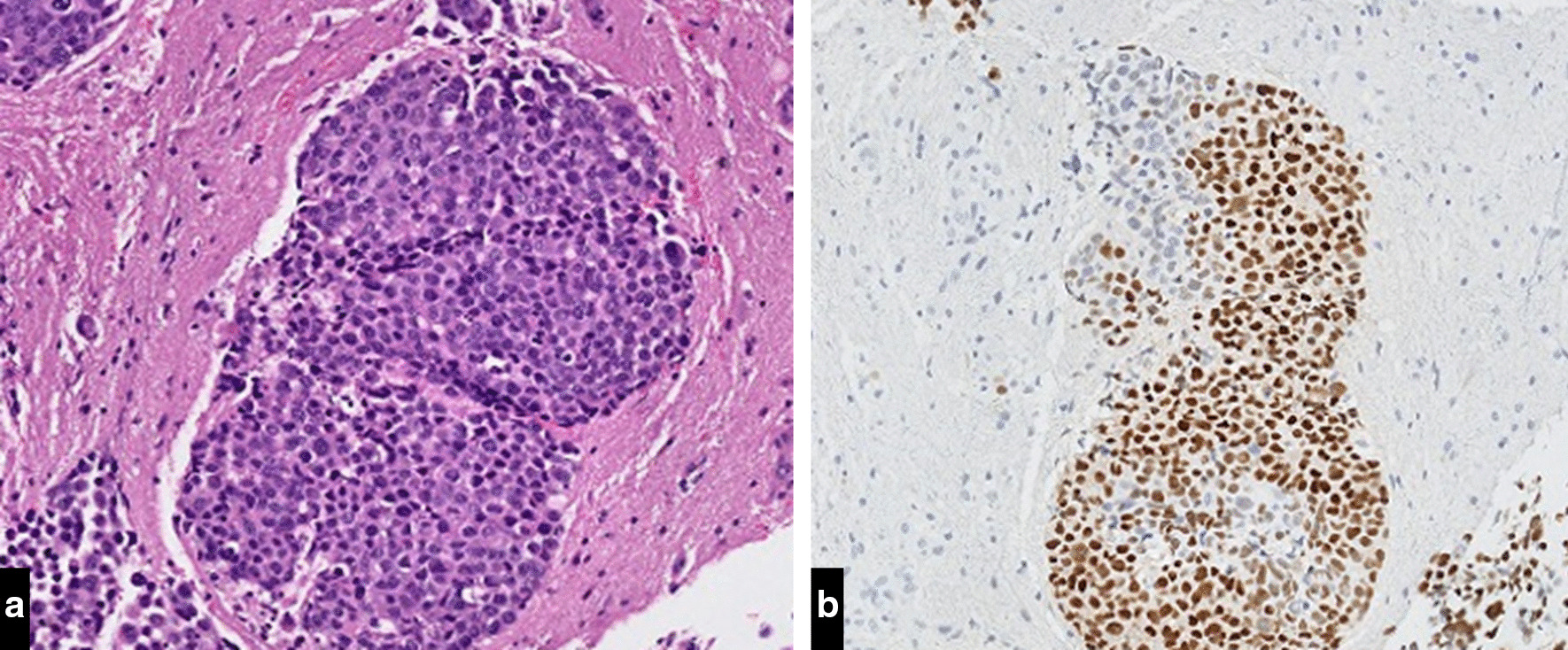
**a** Metastatic breast carcinoma (left thalamic brain mass). H&E stain, ×50. **b** Expression of Estrogen Receptors (left thalamic brain mass), IHC stain, ×50

**Fig. 4:**
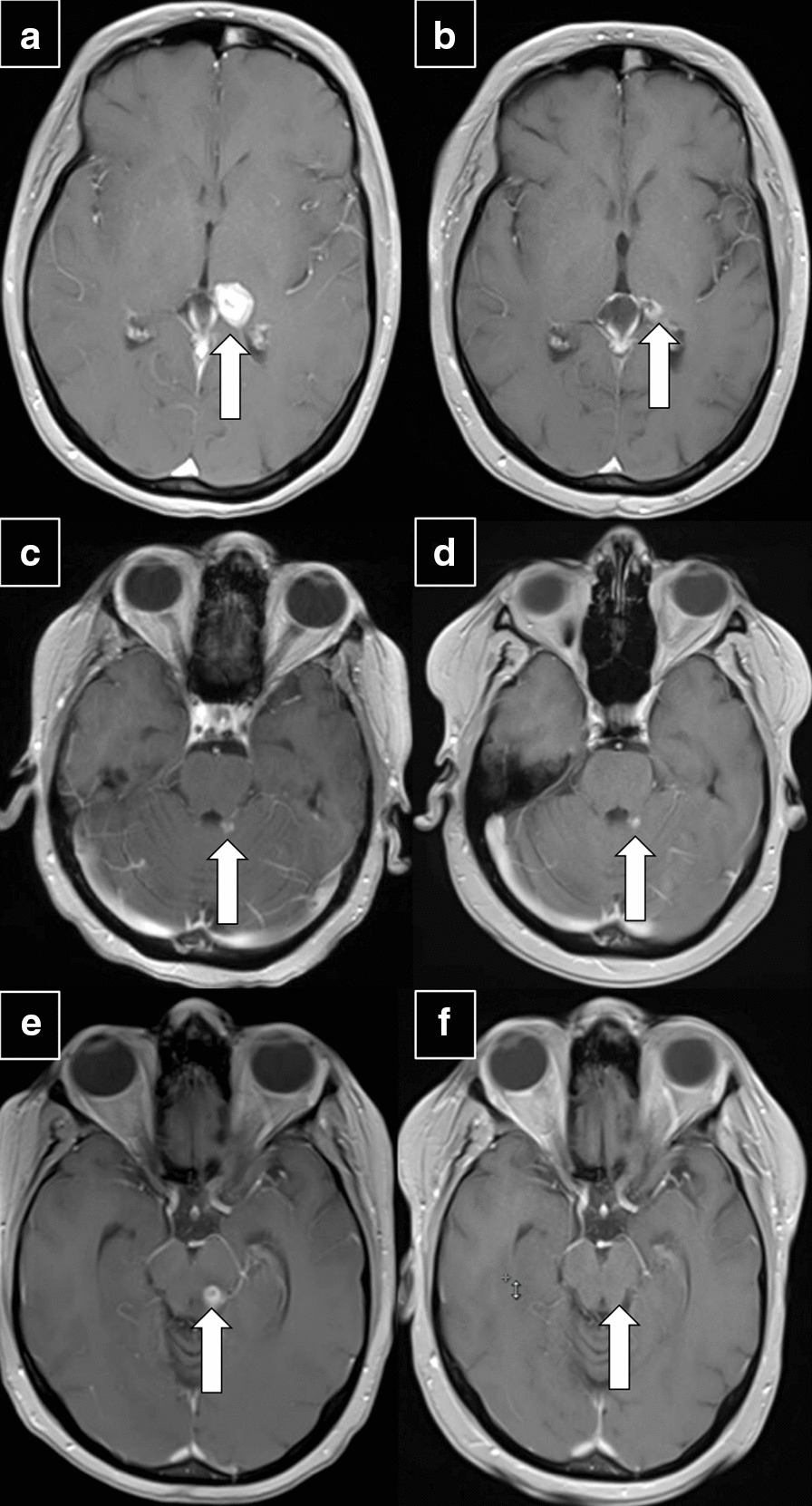
T1-weighted, post-contrast MRI brain images of a: **a** left thalamic lesion in July 2014 (arrow) treated with fractionated stereotactic radiotherapy (3000 cGy in 5 fractions) and **b** post-treatment images in August 2015; **c** small contrast-enhancing lesion in the left cerebellar hemisphere (arrow) in December 2014 which was treated with stereotactic radiation to the left cerebellar lesion in January 2015 and **d** post-treatment images in August 2015; **e** a 1.3 × 1.0 × 1.9 cm lesion involving the posterior inferior left thalamus (arrow) extending inferiorly to involve the left midbrain tectum in March 2015 treated with whole brain radiation therapy with 30 Gy over 10 fractions completed on April 2015 and **f** post-treatment images in August 2015.

The patient was well until approximately 3 years (July 2014) after the initial diagnosis, when she began to experience right-sided headaches, right temple numbness and syncopal episodes. Her neurological examination was significant for 4/5 reduced motor strength in the left upper and lower extremities. Magnetic resonance imaging (MRI) of the brain showed an enhancing mass in the left thalamus measuring 1.6 cm in diameter (Fig. 4a). Computed tomography scan of chest, abdomen and pelvis with contrast and whole-body nuclear bone scan showed no evidence of extra-cranial metastases. She then underwent a biopsy of the left thalamic mass under MRI guidance which revealed metastatic breast cancer (Fig. 3a) that was 80% positive for ER (Fig. 3b), 15–20% positive for PR and negative for HER2 (IHC 0, HER2/CEP17:1.34). Circulating CA15-3N and CA27.29 levels were measured at the time of the diagnosis of metastatic disease and were within normal levels (21.5 U/mL and 13.7 U/mL respectively). She subsequently received fractionated stereotactic radiotherapy (3000 cGy in 5 fractions) to the left thalamus. She had resolution of her symptoms and was started on exemestane with GnRH agonist goserelin. Goserelin was discontinued after the patient underwent bilateral oophorectomy due to concerns of inadequate ovarian suppression.

The patient continued to do well until a follow up brain MRI in December 2014 demonstrated a new focus of enhancement in the left cerebellar hemisphere measuring 5 × 5 mm (Fig. 4c). She was treated with stereotactic radiation therapy to the left cerebellar lesion. Unfortunately, brain MRI in March 2015 demonstrated interval enlargement of the enhancing lesion involving the left thalamus now measuring 1.9 cm. She therefore underwent whole brain radiation therapy with 30 Gy over 10 fractions (Fig. 4e). She did well without evidence of disease progression and neurological deficits with stable MRI scans (Fig. 4b, d, f) until April 2016, when she was found to have worsening gait, dizziness and tremor. Brain MRI in May 2016 demonstrated progression of the left cerebellar lesion for which she underwent posterior fossa craniotomy that revealed necrotic debris, calcifications, granulation tissue and hemosiderin-laden macrophages consistent with post-radiation treatment effects and without any viable tumor. Unfortunately, the patient’s functional status continued to deteriorate and in August 2016 she presented with status epilepticus. Brain MRI did not show any evidence of disease progression. She refused lumbar puncture for evaluation of leptomeningeal carcinomatosis. Despite adjustment of her antiepileptic medications, she was admitted multiple times with refractory seizures. Of importance, all staging scans up to this point have not demonstrated any evidence of metastatic disease outside of the CNS. She elected to pursue hospice care and passed away soon after in December 2016.

## Discussion

Brain metastases in patients with breast cancer are associated with poor prognosis and diminished quality of life. One year survival rate of patients with metastatic breast cancer to the brain was reported as only ~ 20% [7]. Several risk factors associated with increased brain metastases have been identified, including younger age, poorly differentiated tumors (high grade), HR negative status and metastases to four or more axillary lymph nodes at the time of diagnosis [8]. The HR positive breast cancers were significantly associated with bone metastases and were less frequently observed in patients with lung and brain metastases. In contrast, patients with HER2-positive and triple negative breast cancer had a 5.3-fold increased risk of developing brain metastasis compared with those with HR positive, HER2-negative disease [9]. Furthermore, HR discordance in primary tumor and brain tumor is seen in 18-54% of cases [10, 11]. The reason for the discordant HR status is not clearly understood but one explanation may be genomic tumor heterogeneity that leads to treatment induced selection of resistant clones with altered hormone receptor expression [12]. A retrospective study by Lindström *et al.* showed that discordant cases had worse survival [13]. whereas a prospective trial by Amir *et al.* and retrospective trial Qi Shen *et al.* failed to demonstrate adverse effects on clinical outcome [10, 14]. Our patient had ER/PR expression and lack of HER2 over-expression in both primary tumor and metastatic brain lesion.

Solitary brain metastasis has been reported in approximately 0.8–14% in patients with breast cancer across different data sets [15, 16]. HER2 positive breast cancer patients are more likely to develop solitary brain metastasis without systemic relapse [17]. Older age (≥ 40 years) at diagnosis of breast cancer (*p* = .04), larger tumor size (T2 and T3; *p* = .002), advanced baseline stage (III and IV; *p* < .0001), and HR positive HER2 positive subtype (*p* = .01) were more frequently found in patients who developed brain metastases as the first recurrence compared with those who had a first recurrence at other sites in the body [10]. In the case of HR positive breast cancer patients, time to brain metastasis is longer (55 months) with a better median overall survival (OS) of about 10–23 months as compared to TNBC (time to metastasis: 27.5 months; median OS: 3–7 months). HER2 positive patients, on average, have intermediate time until brain metastasis (34–47 months), depending on ER negative or ER positive histologic status, respectively, and a median OS from time of diagnosis of brain metastases of about 17.9 months [17]. Therefore ER, PR and HER2 expression status impact both the time from initial diagnosis of metastasis to the brain as well as median survival following this diagnosis. In retrospective survey of 420 metastatic breast cancer patients with brain metastasis, patients who survived more than 18 months had younger age, were premenopausal, and had solitary brain metastasis or HR positive status. Our index patient had all four characteristics and lived approximately 28 months after first diagnosis of brain metastasis [15].

This case report describes a rare pre-menopausal patient with history of operable, HR positive, HER2 negative breast cancer who experienced relapse of her disease in the form of a single metastatic lesion to the left thalamus. Over time, her metastatic disease in the brain worsened but she continued to show no radiographic evidence of metastatic disease outside of the central nervous system. Whether this isolated brain recurrence can be due to CNS serving as a sanctuary site for cancer cells which could not be killed by prior chemotherapy due to blood brain barrier or whether brain was the first site of disseminated metastatic disease with remaining sites having only microscopic tumor deposits is unclear. Few studies to date have been able to shed a light on this issue. After reviewing the literature, we found only one single institutional, retrospective analysis of 128 breast cancer patients with brain metastases as the first and isolated site of recurrence. This analysis revealed that 42.1 % of these patients (*N *= 54) had only one metastatic brain lesion. Of those patients, 28% were HR positive and HER2 negative, while 37% and 35% were TNBC and HER2 positive respectively [18]. In this study factors that impacted poorer patient survival were Karnofsky performance status of < 70, more than 1 metastatic brain lesion, presence of leptomeningeal disease and non-receipt of systemic therapy after diagnosis of metastatic disease. Indeed, patients who did not receive systemic therapy had considerably poorer survival of only 4 months compared to survival of 15 months for patients treated with systemic therapy (*p *< 0.001). This finding suggests that perhaps patients with radiographic evidence of isolated brain metastases could have more widely disseminated microscopic disease that is undetectable on staging scans. Management of breast cancer patients with brain only metastases is very challenging as very few studies have been conducted to provide evidence for efficacy and safety of systemic treatments for this clinical scenario. In fact, the overwhelming majority of therapeutic clinical trials excluded patients with progressive CNS metastases due to poor prognosis and this resulted in paucity of data supporting or refuting use of many otherwise effective therapeutic options for breast cancer. Complicating the problem is the fact that most cytotoxic agents are unable to cross the blood brain barrier and therefore are expected to have limited activity in controlling brain metastases. Yet, there are a few studies performed in women with breast cancer and CNS metastases suggesting that drugs with smaller molecular weight (such as capecitabine, cyclophosphamide or liposomal doxorubicin as well as small molecule tyrosine inhibitors) can produce limited responses in the CNS and therefore are likely to penetrate the brain parenchyma at least to some extent [19–21]. Furthermore, intrathecal therapy can be beneficial although its limited activity has to be carefully weighed against its multiple toxicities and risks [22]. In melanoma, a few studies have demonstrated benefit of combining brain radiation and immune checkpoint inhibitors, although studies in patients with breast cancer have not been performed [23]. No prospective randomized trials have been conducted to evaluate the role of systemic therapy (whether endocrine, targeted or cytotoxic agents) following resection and/or stereotactic radiation therapy of solitary brain metastasis. Whether use of systemic therapy in this setting would decrease the probability of disease progression and improve survival is an important yet unanswered question. Single institution retrospective study has suggested a beneficial role of systemic chemotherapy following local therapy for brain metastases in patients with breast cancer and no evidence of disease outside of the CNS. [18] Prospective studies to sort this out and establish most effective treatment approaches to patients with isolated CNS metastases will be challenging due to a small incidence of solitary brain metastases in breast cancer patients.

Taken together, we strongly feel that eligibility criteria of clinical trials that have been studying novel and promising agents for breast cancer need to become more permissive to allow enrollment patients with progressive CNS disease as this clinical setting is one of the most challenging problems for oncologists to treat and therefore represents a highly unmet need.

## Conclusion

This report describes a unique case of a pre-menopausal patient with operable, HR positive, HER2 negative breast cancer who developed CNS only recurrence of her disease. Despite eventual disease progression within the CNS, she has never developed extra-cranial metastatic disease. This emphasizes the need for clinical suspicion in patients with treated, operable hormone receptor positive, HER2 negative breast cancer who present with neurological symptoms. This case report suggests that patients with CNS only metastatic disease have the potential to experience long survival if their CNS disease is treated with aggressive local and systemic therapy. However, large prospective study is needed to further investigate the outcome in patients with hormone receptors positive and Her 2 negative breast cancer treated with systemic therapy vs combination of systemic therapy and local therapy.

## Data Availability

The datasets used and/or analyzed during the current study are available from the corresponding author on reasonable request.

## Abbreviations

CNS: Central Nervous System
ER: Estrogen Receptor
HER2: Human Epidermal growth Factor Receptor 2
HR: Hormonal Receptors
IHC: Immunohistochemical
MRI: Magnetic Resonance Imaging
PR: Progesterone Receptor
TNBC: Triple Negative Breast cancer
